# Circle of Willis variation in a complex stroke presentation: a case report

**DOI:** 10.1186/1471-2377-6-13

**Published:** 2006-03-15

**Authors:** Hedley CA Emsley, Carolyn A Young, Richard P White

**Affiliations:** 1 Division of Neuroscience, University of Liverpool, The Walton Centre for Neurology & Neurosurgery, Lower Lane, Liverpool L9 7LJ, UK; 2 The Walton Centre for Neurology & Neurosurgery, Liverpool, UK

## Abstract

**Background:**

The impact of circle of Willis anatomical variation upon the presentation of stroke is probably underrecognised.

**Case presentation:**

A 63-year-old right-handed woman developed a left hemiparesis and right leg weakness sequentially following a road traffic accident (RTA). Despite initial concern about the possibility of cervical spinal cord injury, the final diagnosis was bilateral artery-to-artery embolic cerebral infarction with dominant right internal carotid artery.

**Conclusion:**

The case illustrates the complex presentation of stroke as a pseudo-cervical cord lesion and the impact of circle of Willis anatomical variation upon the expression of large vessel cerebrovascular disease.

## Background

Bilateral artery-to-artery embolic cerebral infarction attributable to circle of Willis anatomical variation is described here as an initially unexpected cause of progressive triplegia which occurred in close temporal proximity to a road traffic accident, where traumatic cervical cord injury was initially inferred. The diagnosis of stroke is often not straightforward and this case underlines the need for early, detailed neurological evaluation.

## Case presentation

### Case report

A 63-year-old right-handed woman attended her local hospital with a left hemiparesis of sudden onset 24 hours after a RTA. Her motor vehicle had collided with a parked vehicle. There was neither loss of consciousness nor any definite head or whiplash injury. Within a further 24 hours she developed right leg weakness. Computed tomography (CT) brain scan revealed a small low-density area in the right fronto-parietal region only; a CT scan of her cervical spine was normal. Given her progressive triplegia, immediate transfer to our centre was made, to exclude cervical spinal cord injury.

Initial assessment revealed her to be orientated with fluent speech. However, she had evidence of cognitive impairment with an inappropriate jovial affect, poor cognitive estimates and concrete thinking, bradyphrenia and was unable to provide a reliable history. Additional history was obtained from her husband and family. There was no preceding history of cognitive decline but some reduced self-care and hygiene, weight loss of approximately 8 kilograms over three months, and altered bowel habit. Her family had witnessed three episodes of left arm monoparesis, lasting between minutes and 1–2 hours, in the 48 hours preceding her accident. Twenty-four hours after her accident she developed a dense left hemiparesis of sudden onset.

She consumed less than 2 units of alcohol per week, was an ex-smoker, and had no other past medical history of note. She had a family history of ischaemic heart disease.

General examination was unremarkable and she was in sinus rhythm. Neurological examination revealed impaired volitional saccades to the left, but normal targeted saccades. Snout and palmomental reflexes were present. Mild left sided visuospatial dysfunction was present. No sensory inattention was demonstrated. She had a dense left hemiparesis (MRC grade 0 arm and leg) and mild right leg monoparesis (MRC grade 4). Deep tendon reflexes were brisk bilaterally, and both plantar responses were extensor. There was no sensory level.

Formal neuropsychological assessment later confirmed specific difficulties with visual memory, executive functioning and perceptual/constructional abilities.

Urgent brain and cervical spine MRI, revealed an ischaemic lesion in the right fronto-parietal region but no structural cervical cord pathology. Diffusion weighted images were not obtained. A repeat MRI brain scan, 8 days after the onset of left hemiparesis showed additional infarction in the distal left anterior cerebral artery territory.

**Figure 1:**
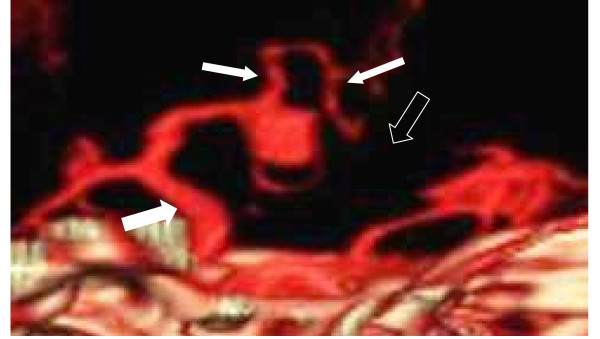
3D-CTA image of the circle of Willis. The A1 segment of the left anterior cerebral artery is absent (open arrow). The ICA on the right (large solid arrow) is feeding both anterior cerebral arteries (small solid arrows) and the ipsilateral middle cerebral artery. In addition there is an anterior communicating artery aneurysm.

Comprehensive serial blood tests revealed the following abnormal results (normal ranges and units in brackets): white blood cell count between 11.6 and 13.9 (4 to 11 × 10^9^/L), C-reactive protein between 17.8 and 49.5 (< 8 mg/L), erythrocyte sedimentation rate between 54 and 65 (5 to 15 mm/h), total serum cholesterol 5.7 (<5 mmol/L), carcinoembryonic antigen (CEA) 15.7 (0 to 3.5 ng/mL). Carotid ultrasound examination revealed a right internal carotid artery (ICA) stenosis >85%, whilst all other vessels were normal. Routine cerebrospinal fluid examination was normal. CTA neck confirmed the right ICA stenosis, but no significant left ICA disease. CTA circle of Willis (CoW) showed the right A1 anterior cerebral artery (ACA) segment to be dominant, the left A1 ACA segment to be absent, and an anterior communicating artery (AcoA) aneurysm (Figure 1). The remainder of the CoW was considered to be normal. A transthoracic echocardiogram demonstrated no cardiac embolic source. A colonoscopy revealed a rectal tumour, confirmed on biopsy to be a moderately differentiated adenocarcinoma.

Pharmacological treatment included aspirin, pravastatin and perindopril. She also received care from a multi-disciplinary rehabilitation team including physiotherapy. By 10 days after the onset of the left hemiparesis, improvement was noted in all of her deficits, and by 18 days she was regaining power in the left hand and wrist. She was transferred back to her referring hospital for management of her rectal tumour.

The neurological formulation at discharge was one of symptomatic right internal carotid artery stenosis and embolic stroke. It is possible that the RTA was a complication of left-sided visuospatial dysfunction. The subsequent presentation is consistent with multiple bilateral embolic strokes, secondary to CoW variation.

## Discussion

We report successive bilateral anterior circulation cerebral infarctions in a 63-year-old in close temporal relation to a RTA. The triplegia initially led to the suspicion of cervical spinal cord injury at her local hospital, but the history of probable TIA's prior to the RTA and subsequent detailed clinical evaluation and investigation established a clear cerebrovascular aetiology. A coincidental rectal adenocarcinoma is most likely to have accounted for elevated tumour and inflammatory markers found during the course of investigation.

Delayed presentation of cervical cord injury following trauma is well described and may progress up to 48 hours following injury [1]. This patient's triparesis and examination findings were considered to suggest this by the referring hospital. However, the absence of other clinical features such as head or neck pain, segmental neurological motor or sensory level, or bladder dysfunction, might have pointed to an alternative diagnosis at an early stage.

The demonstration of a dominant A1 segment of the right ACA together an absent left A1 ACA segment suggests that in this patient both A2 ACA segments received their supply from the right ICA. Anatomical variation of the ACA, particularly in association with AcoA aneurysms is recognised [2]. We consider it most likely that the infarction in the left ACA territory occurred due to artery-to-artery thromboembolism from the severe right ICA stenosis. Although transhemispheric cerebral diaschisis has been proposed as an explanation for symptoms occurring in the contralateral hemisphere following stroke [3], the majority of ACA territory infarcts are embolic in aetiology [4]. It is unusual to be able to document crossed embolic phemonena such as this.

Other potential stroke mechanisms to be considered in this case include carotid artery dissection, which is known to occur either spontaneously [5] or in the context of trauma [6]. However, given that the symptoms in this patient began prior to the RTA, carotid artery dissection is not likely to have been the explanation in this case. Furthermore, no associated features, which might be expected such as, pain or oculosympathetic palsy ipsilateral to the injured ICA were observed here, nor did the imaging findings support arterial dissection.

Although spontaneous thrombosis of an unruptured AcoA aneurysm is described [7], embolisation from an unruptured intracranial aneurysm sac is an unusual cause of ischaemic stroke [8]. With the alternative embolic source in this patient, the AcoA aneurysm is much more likely to have been incidental, especially given the initial right parietal lobe infarct.

## Conclusion

This case raises several important points highlighting the value of careful clinical assessment, and underlines the importance of early, detailed neurological evaluation. Furthermore, we have been able to demonstrate the likely mechanism for bilateral cerebral infarction in this case and the impact of circle of Willis anatomical variation upon the expression of large vessel cerebrovascular disease.

## Competing interests

The author(s) declare that they have no competing interests.

## Authors' contributions

All authors conceived of the study. HCAE drafted the report, CAY and RPW assisted in its writing. All authors read and approved the final manuscript.

## Pre-publication history

The pre-publication history for this paper can be accessed here:



## References

[B1] Mahale YJ, Silver JR (1992). Progressive paralysis after bilateral facet dislocation of the cervical spine. J Bone Joint Surg Br.

[B2] Perlmutter D, Rhoton AL (1976). Microsurgical anatomy of the anterior cerebral-anterior communicating-recurrent artery complex. J Neurosurg.

[B3] Gonzalez Aguado E, Marti Fabregas J, Marti Vilalta JL (2000). The phenomenon of diaschisis in cerebral vascular disease. Rev Neurol.

[B4] Bogousslavsky J, Regli F (1990). Anterior cerebral artery territory infarction in the Lausanne Stroke Registry: clinical and etiologic patterns. Arch Neurol.

[B5] Schievink WI (2001). Spontaneous dissection of the carotid and vertebral arteries. N Engl J Med.

[B6] Rommel O, Niedeggen A, Tegenthoff M, Kiwitt P, Botel U, Malin J (1999). Carotid and vertebral artery injury following severe head or cervical spine trauma. Cerebrovasc Dis.

[B7] Brownlee RD, Tranmer BI, Sevick RJ, Karmy G, Curry BJ (1995). Spontaneous thrombosis of an unruptured anterior communicating artery aneurysm. An unusual cause of ischemic stroke. Stroke.

[B8] Qureshi AI, Mohammad Y, Yahia AM, Luft AR, Sharma M, Tamargo RJ, Frankel MR (2000). Ischemic events associated with unruptured intracranial aneurysms: multicenter clinical study and review of the literature. Neurosurgery.

